# Characterization of NGFFYamide Signaling in Starfish Reveals Roles in Regulation of Feeding Behavior and Locomotory Systems

**DOI:** 10.3389/fendo.2018.00507

**Published:** 2018-09-19

**Authors:** Ana B. Tinoco, Dean C. Semmens, Emma C. Patching, Elizabeth F. Gunner, Michaela Egertová, Maurice R. Elphick

**Affiliations:** School of Biological and Chemical Sciences, Queen Mary University of London, London, United Kingdom

**Keywords:** echinoderm, neuropeptide S, crustacean cardioactive peptide, NGFFYamide, NGFFYamide receptor, tube feet, locomotion, feeding

## Abstract

Neuropeptides in deuterostomian invertebrates that have an Asn-Gly motif (NG peptides) have been identified as orthologs of vertebrate neuropeptide-S (NPS)-type peptides and protostomian crustacean cardioactive peptide (CCAP)-type neuropeptides. To obtain new insights into the physiological roles of NG peptides in deuterostomian invertebrates, here we have characterized the NG peptide signaling system in an echinoderm—the starfish *Asterias rubens*. The neuropeptide NGFFYamide was identified as the ligand for an *A. rubens* NPS/CCAP-type receptor, providing further confirmation that NG peptides are orthologs of NPS/CCAP-type neuropeptides. Using mRNA *in situ* hybridization, cells expressing the NGFFYamide precursor transcript were revealed in the radial nerve cords, circumoral nerve ring, coelomic epithelium, apical muscle, body wall, stomach, and tube feet of *A. rubens*, indicating that NGFFYamide may have a variety of physiological roles in starfish. One of the most remarkable aspects of starfish biology is their feeding behavior, where the stomach is everted out of the mouth over the soft tissue of prey. Previously, we reported that NGFFYamide triggers retraction of the everted stomach in *A. rubens* and here we show that *in vivo* injection of NGFFYamide causes a significant delay in the onset of feeding on prey. To investigate roles in regulating other aspects of starfish physiology, we examined the *in vitro* effects of NGFFYamide and found that it causes relaxation of acetylcholine-contracted apical muscle preparations and induction of tonic and phasic contraction of tube feet. Furthermore, analysis of the effects of *in vivo* injection of NGFFYamide on starfish locomotor activity revealed that it causes a significant reduction in mean velocity and distance traveled. Interestingly, experimental studies on mammals have revealed that NPS is an anxiolytic that suppresses appetite and induces hyperactivity in mammals. Our characterization of the actions of NGFFYamide in starfish indicates that NPS/NG peptide/CCAP-type signaling is an evolutionarily ancient regulator of feeding and locomotion.

## Introduction

Neuropeptides are intercellular signaling molecules that are secreted by neurons to regulate a diverse range of physiological processes and behaviors in animals (1, 2). They typically exert their effects on target cells by binding to and activating cognate G-protein coupled receptors (GPCRs) belonging to the rhodopsin-β, rhodopsin-γ, and secretin-type receptor families (3).

Phylogenetic analysis has revealed that the evolutionary origin of at least 30 neuropeptide signaling systems can be traced back to the bilaterian common ancestor of deuterostomes and protostomes (4–6). Interestingly, the amino acid sequences of some neuropeptides are highly conserved throughout the Bilateria, whereas other neuropeptides have diverged to such an extent that they are not immediately recognizable as orthologs based on their amino acid sequence. An example of the former are vasopressin/oxytocin (VP/OT)-type neuropeptides, which are typically C-terminally amidated cyclic nonapeptides (7). An example of the latter are a family of neuropeptides that are paralogs of VP/OT-type neuropeptides, which include neuropeptide-S (NPS) in vertebrates, crustacean cardioactive peptide (CCAP) in protostomes and NG peptides in invertebrate deuterostomes (e.g., NGFFYamide, which is the focus of this paper) (8).

NPS is a 20-residue neuropeptide (SFRNGVGTGMKKTSFQRAKS) that was first discovered in rodents (9) and has subsequently been identified throughout the tetrapod vertebrates (10). NPS induces arousal and anxiolytic-like effects in rodents (9, 11, 12), whilst polymorphisms in the NPS receptor (NPSR) are associated with panic disorders in humans (13, 14). The NPS/NPSR system also regulates other physiological/behavioral processes in mammals, including locomotion (15–19), gastrointestinal functions (20, 21) and feeding (15, 18, 22).

Orthologs of the NPSR are present in arthropods and other protostomian invertebrates (4, 5) and these are activated by CCAP-type neuropeptides (23, 24), which do not exhibit sequence similarity with NPS. CCAP is a 9-residue neuropeptide (PFCNAFTGC-NH_2_) that was first discovered in the crustacean *Carcinus maenas* (25), with CCAP-type neuropeptides subsequently identified in other protostomian invertebrates (4, 5). As its name implies, CCAP was initially discovered on account of its cardioacceleratory effects (25), but it has since been found to have a key role in neural control of ecdysis in crustaceans (26, 27) and insects (28–31). Furthermore, experimental studies on the cockroach *Periplaneta americana* have revealed that CCAP also regulates gastrointestinal activity (32), feeding and locomotion (33).

Important insights into the evolutionary relationship between NPS-type neuropeptides in vertebrates and CCAP-type neuropeptides in protostomes have been provided by the discovery of the NG peptide signaling system in invertebrate deuterostomes, which occupy an intermediate phylogenetic position with respect to vertebrates and protostomes. Members of this neuropeptide family have a characteristic Asn-Gly (NG) motif (34), which provided the basis for the name “NG peptide”. Sequencing of the genome of the sea urchin *Strongylocentrotus purpuratus* (Phylum Echinodermata) first enabled the discovery of a gene encoding an NG peptide precursor protein, which contains two tandem copies of the neuropeptide NGFFFamide (NGFFF-NH_2_) (35). Furthermore, the NGFFFamide precursor was found to have a C-terminal neurophysin domain, which was a surprising finding because hitherto neurophysins were thought to be uniquely associated with the precursors of VP/OT-type neuropeptides (36, 37). Subsequently, NG peptide precursors have been identified in other echinoderm species (8, 38–40), the hemichordate *Saccoglossus kowalevskii* and the cephalochordate *Branchiostoma floridae* (34). This has revealed that the presence of a C-terminal neurophysin domain is a conserved feature of NG peptide precursors, although the neurophysin domain has been lost in holothurian (sea cucumber) NG peptide precursors.

Evidence that NG peptides are orthologs of vertebrate NPS-type neuropeptides was provided by analysis of the sequence of the NG peptide precursor in the cephalochordate *B. floridae*, which comprises two copies of a peptide (SFRNGVG) that is identical to the N-terminal region of NPS (SFRNGVGTGMKKTSFQRAKS) (34). However, definitive proof was provided with the discovery that an NPS/CCAP-type receptor in the sea urchin *S. purpuratus* is activated by the neuropeptide NGFFFamide (8). Thus, a bilaterian neuropeptide family comprising NPS in vertebrates, NG peptides in invertebrate deuterostomes and CCAP-type neuropeptides in protostomes was unified. Furthermore, discovery and characterization of NG peptide signaling in deuterostomes provided a key missing link for reconstructing the evolutionary history of the paralogous VP/OT-type and NPS/CCAP-type neuropeptide signaling systems. It can be inferred that the presence of a neurophysin domain was an ancestral characteristic of the precursors of both neuropeptide families, which has then been retained in all bilaterian VP/OT-type precursors and in the NG peptide precursors of invertebrate deuterostomes but which has been lost independently in vertebrate NPS-type precursors and in protostome CCAP-type precursors (5, 8, 41).

The discovery that NG peptides are orthologs of NPS and CCAP-type neuropeptides has provided an interesting context for investigating the physiological roles of NG peptides in invertebrate deuterostomes. Currently, nothing is known about the physiological roles of NG peptides in cephalochordates or hemichordates; however, progress has been made in examining NG peptide function in echinoderms. The first NG peptide to be discovered was the myoactive neuropeptide NGIWYamide, which causes contraction of the longitudinal body wall muscle in the sea cucumber *Apostichopus japonicus* (42, 43). Subsequently, it has been found that NGIWYamide also acts as a gonadotropic neuropeptide, triggering oocyte maturation and spawning in *A. japonicus* (44). Discovery of the NGFFFamide precursor in *S. purpuratus* facilitated investigation of the pharmacological actions of NGFFFamide in sea urchins, which revealed that it causes contraction of tube foot and esophagus preparations from the sea urchin *Echinus esculentus* (35). Thus, this finding was consistent with the myotropic action of NGIWYamide in sea cucumbers.

Investigation of the physiological roles of NG peptides in echinoderms was advanced most recently with the discovery of the precursor of the NG peptide NGFFYamide (NGFFY-NH_2_) in the starfish *A. rubens*. Research on neuropeptide systems in this species led to the discovery of the first echinoderm neuropeptides to be sequenced—the SALMFamide neuropeptides S1 and S2 (45). S1 and S2 act as muscle relaxants in starfish and, more specifically, *in vitro* and *in vivo* pharmacological experiments revealed that S1 and S2 trigger relaxation and eversion of the stomach in *A. rubens* (46, 47). This was of interest because stomach eversion occurs naturally in starfish when they feed extra-orally on prey (e.g., mussels). Therefore, it was postulated that when starfish feed, S1 and S2 may be released to enable relaxation and eversion of the stomach out of the mouth and over the digestible tissue of prey (46, 47). Conversely, the existence of a counteracting neuropeptide that triggers stomach contraction and retraction when feeding is completed was also postulated. Because NG peptides act as muscle contractants in sea cucumbers and sea urchins, the starfish neuropeptide NGFFYamide was tested on starfish and found to cause stomach contraction *in vitro* and stomach retraction *in vivo* (39).

Having discovered, quite specifically, that NGFFYamide acts to cause stomach contraction and retraction in starfish (39), the aim of this study was to characterize more generally the NGFFYamide signaling system in starfish and this has been accomplished using a variety of experimental approaches. Firstly, an *in vitro* cell-based assay was employed to demonstrate that NGFFYamide is the ligand for an *A. rubens* NPS/CCAP-type receptor that was identified previously as a candidate receptor for this neuropeptide (8). Secondly, mRNA *in situ* hybridization methods were employed to investigate the anatomy of NGFFYamide precursor expression in *A. rubens*. Thirdly, informed by the anatomical expression pattern of the NGFFYamide precursor, the *in vitro* pharmacological actions of NGFFYamide on apical muscle and tube feet preparations from *A. rubens* were investigated. Fourthly, and informed by the *in vitro* and *in vivo* actions of NGFFYamide, effects of *in vivo* injection of NGFFYamide on starfish feeding behavior and locomotor activity were examined. Collectively, the findings of this study provide important new insights into the comparative physiology of NPS/NG peptide/CCAP-type neuropeptide signaling in the animal kingdom.

## Materials and methods

### Animals

Adult starfish (*A. rubens*) were collected at low tide from a location near Margate (Kent, UK) or were obtained from a fisherman based at Whitstable (Kent, UK). The starfish were maintained in an aquarium with circulating artificial seawater (Tropical Marine Centre^TM^ Premium Reef-Salt) at a salinity of 32 ‰ and at a temperature of approximately 11°C under a 12-h light/dark cycle. Animals were fed on mussels (*Mytilus edulis*) that were also collected from a location near Margate (Kent, UK) at low tide.

### Pharmacological characterization of the NGFFYamide receptor

We have previously reported the identification of an *A. rubens* NPS/CCAP-type receptor (GenBank accession number: KP171535) that is a candidate receptor for NGFFYamide (8). Here we performed a more comprehensive phylogenetic analysis to investigate the relationship of the *A. rubens* NPS/CCAP-type receptor with NPS/CCAP-type receptors that have been identified and, in some cases, pharmacologically characterized in other species. Phylogenetic analysis was performed using a maximum likelihood method, with sequences aligned using the MUSCLE plugin in MEGA 7 (iterative, 10 iterations, UPGMB as clustering method) (48, 49). The maximum-likelihood tree was built using PhyML version 3.0 (1000 bootstrap replicates, LG substitution model) (50) and edited using FigTree v1.4.2. (http://tree.bio.ed.ac.uk/software/figtree/).

To enable pharmacological characterization of the *A. rubens* NPS/CCAP-type receptor, a pUC57 vector comprising the coding sequence of the receptor was custom synthesized (GenScript®), with a partial Kozak sequence (ACC) incorporated immediately preceding the start codon to optimize the initiation of translation. The full-length sequence was then sub-cloned into a pcDNA3.1^TM^ (+) mammalian expression vector using the blunt-end restriction endonuclease EcoRV. A plasmid maxiprep was subsequently performed using the QIAGEN Plasmid Maxi Kit (QIAGEN). To determine whether synthetic NGFFYamide (NGFFY-NH_2_; Peptide Protein Research Ltd.) acts as a ligand for the *A. rubens* NPS/CCAP-type receptor, a receptor deorphanization assay was performed at concentrations ranging from 10^−20^ M to 10^−4^ M using the same methodology as reported recently for deorphanization of *A. rubens* luqin-type receptors (51). Responses were normalized to the maximum response obtained in each experiment (100% activation) and to the response obtained with the vehicle media (0% activation). Concentration-response curves were fitted with a four-parameter curve and EC_50_ values were calculated from concentration-response curves using Prism 6 (GraphPad), which were based on triplicate measurements from at least two independent transfections.

### Localization NGFFYamide precursor expression using mRNA *in situ* hybridization

Cloning of a cDNA encoding the NGFFYamide precursor (GenBank accession number: KC977457) and the subsequent production of digoxigenin-labeled sense and anti-sense RNA probes has been reported previously (52). The methods employed for *(i)* preparation of paraffin-embedded sections of fixed specimens of *A. rubens* and *(ii)* visualization of NGFFYamide precursor transcripts in sections of *A. rubens* were the same as reported recently for analysis of the expression of other neuropeptide precursors in this species (53–55).

### *In vitro* pharmacology

We have previously reported that NGFFYamide causes concentration-dependent contraction of cardiac stomach preparations from *A. rubens* (39). Here, NGFFYamide was tested *in vitro* to investigate its effect on apical muscle and tube foot preparations from *A. rubens*, using the same methods reported previously when testing other neuropeptides (53, 54, 56). Briefly, the apical muscle was dissected from the aboral body wall of starfish arms and cut into segments approximately 1 cm in length and cotton ligatures were tied at each end. Tube foot preparations were obtained by dissecting from starfish arms a small square-shaped piece of ambulacral body wall containing a single tube foot podium and its associated ampullae. Cotton ligatures were tied around the body wall and the tube foot sucker and then the tube foot was scraped with a blunt scalpel blade to partially remove the external epithelial layer to facilitate accessibility of the underlying muscle layer to NGFFYamide when tested. Apical muscle or tube foot preparations were linked to an isotonic force transducer and maintained in a 20 ml aerated organ bath containing circulating artificial sea water at 11°C. Once a stable baseline was reached, acetylcholine (ACh, 10^−6^ M), a known contractant of starfish apical muscle and tube feet (54, 57), was tested to check the viability of preparations and to enable normalization of excitatory effects of NGFFYamide or facilitate detection of relaxing effects of NGFFYamide (46, 56, 58). Synthetic NGFFYamide (prepared in distilled water) was added to the organ bath to achieve concentrations of 10^−6^ M and 10^−5^ M when tested on apical muscle preparations and from 10^−9^-10^−6^ M when tested on tube foot preparations. Each peptide concentration was tested on at least four individual preparations. A relaxant effect of NGFFYamide on apical muscle preparations was expressed as a percentage reversal of the maximal contraction induced by 10^−6^ M ACh. For tube foot preparations, tonic contractions induced by NGFFYamide and the peak amplitude of phasic contractions induced by NGFFYamide were expressed as a percentage of the maximal contraction induced by 10^−6^ M ACh. For phasic contractions, peak amplitude and frequency were quantified over a 5-min recording period and expressed as mean amplitude and mean peaks/minute, respectively. For *in vitro* characterization of the effects of NGFFYamide on tube foot preparations, a concentration-response curve was fitted with a four-parameter curve and the EC_50_ value was calculated based on six measurements. The *in vitro* effect of NGFFYamide on basal tone was analyzed by one-way ANOVA using Bonferroni's multiple comparisons *post-hoc* test. The *in vitro* effect of NGFFYamide on peak frequency and amplitude was analyzed by a Kruskal-Wallis non-parametric test using Dunn's multiple comparisons *post-hoc* test because these data were found to be not normally distributed when analyzed using the Kolmogorov-Smirnov test. All the statistical analyses were carried out using Prism 6 (GraphPad).

### *In vivo* pharmacology

#### Investigation of effects of NGFFYamide on starfish feeding behavior

**Figure 1:**
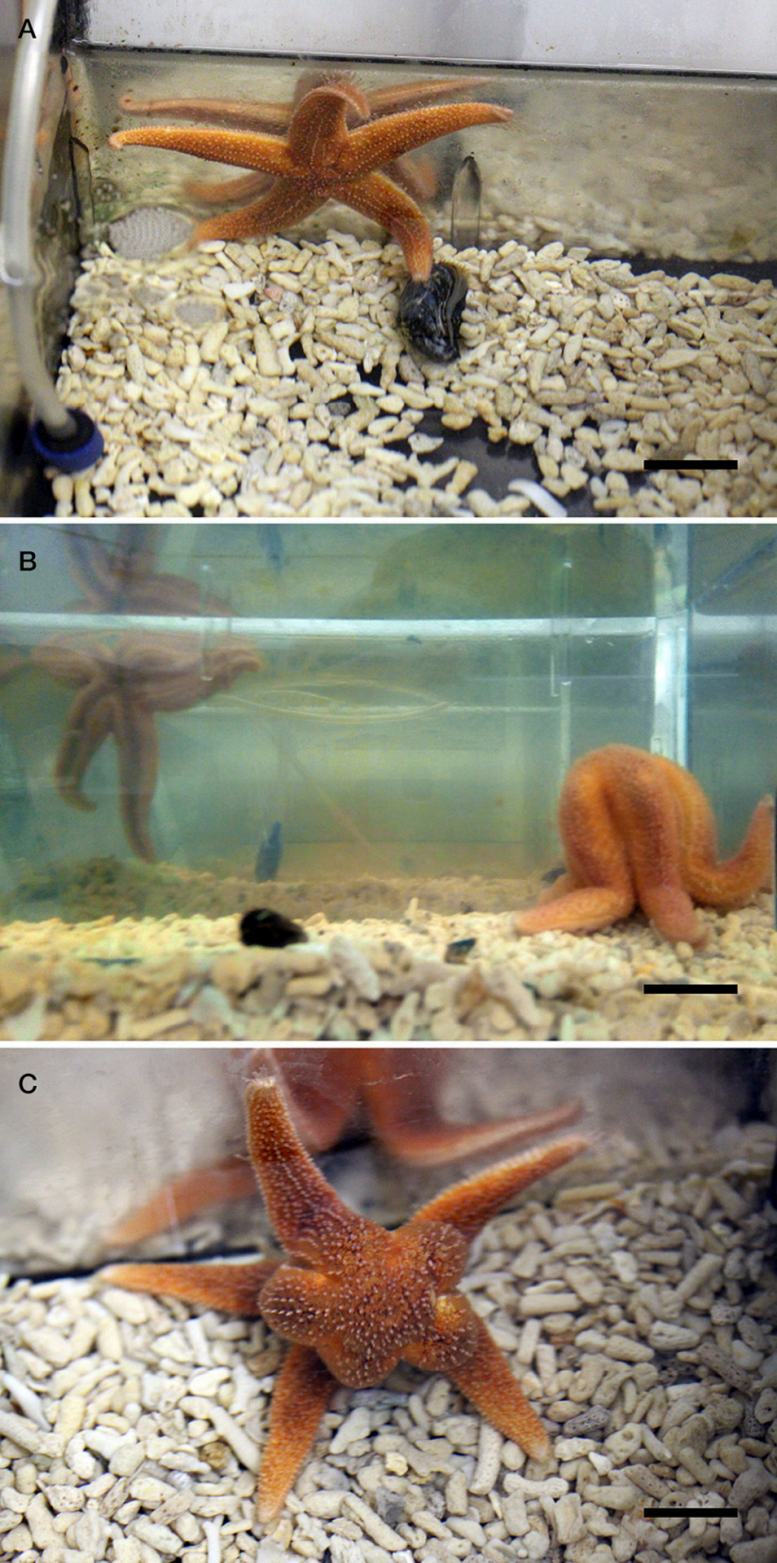
Feeding behavior of the starfish *A. rubens* in a laboratory setting. **(A)** Here a specimen of *A. rubens* can be seen making contact with a mussel (*M. edulis*) in an aquarium tank. In experiments reported in this paper, the time elapsed from when the starfish was placed at the opposite end of the tank to the mussel to when the starfish made contact with the mussel (“time to touch”) was measured and recorded. **(B,C)** Here a specimen of *A. rubens* can be seen enclosing a mussel at the onset of a feeding bout, with photographs taken from a side view **(B)** and from above **(C)**. In experiments reported in this paper, the time elapsed from when the starfish was placed at the opposite end of the tank to the mussel to when the starfish enclosed the mussel (“time to enclose”) was measured and recorded. Scale bars: **(A)**: 2 cm; **(B)**: 2.5 cm; **(C)**: 1.5 cm.

Previously, we reported that NGFFYamide triggers contraction and retraction of the cardiac stomach in *A. rubens*, indicating that this neuropeptide has a physiological role in neural control of the extra-oral feeding behavior of starfish (39). Here, we investigated if *in vivo* injection of NGFFYamide affects the feeding behavior of *A. rubens* on a prey species—the mussel *Mytilus edulis*. For these experiments, 32 adult starfish that met the following criteria were used: *(i)* all five arms were intact, *(ii)* exhibited a normal righting response (59) and *(iii)* after 24 days of starvation, exhibited normal feeding behavior on a mussel. The selected individuals were then maintained in an aquarium with circulating seawater but without food for 24 days. The starfish were then transferred to and kept individually in Plexiglas aquaria (27.5 × 19 × 19.6 cm) containing 6 l of aerated seawater and with the base covered by gravel (Tropical Marine Centre^TM^ Gravel Coarse #5). The exterior of the sides and base of the aquarium tanks was covered with black plastic. These animals were then divided into a control group (to be injected with distilled water) of 15 animals (mean diameter of 11.9 ± 0.3 cm) and a test group (to be injected with NGFFYamide) of 17 animals (mean diameter of 12.5 ± 0.3 cm). After three days of acclimation (27 days of starvation), the starfish were injected with 10 μl of distilled water (control group) or 10 μl of 10^−4^ M synthetic NGFFYamide (test group). This peptide dose was selected for the test group because the volume of the perivisceral coelomic fluid in starfish with a diameter of 11–13 cm is ~10 ml and therefore the estimated final concentration of NGFFYamide in the perivisceral coelom is ~10^−7^ M, which is the concentration at which NGFFYamide was found to have a maximal effect when tested previously on *in vitro* preparations of the cardiac stomach (39). Hamilton 75N 10 μl syringes (Sigma) were used to inject distilled water or NGFFYamide solution into the perivisceral coelom of animals at two opposite sites (5 μl each) in the aboral body wall of arms, proximal to the junction with the central disk region. Care was taken to inject the distilled water/NGFFYamide into the perivisceral coelom and not into the cardiac stomach. Ten minutes after injection, one mussel (25–33 mm) that had been cleaned of any encrusting organisms (60) was placed at one end of the tank approximately 15 cm from the starfish, whilst also ensuring that one arm of the starfish was touching the wall of the opposite end of the tank with the madreporite directed toward the mussel. Starfish behavior was continuously observed for 3 h, followed by observations at 15 min intervals for 2 h and then a final observation 24 h later. The time taken for the starfish to make contact with the mussel (tube feet touching the mussel; time to touch; Figure 1A), the number of attempts to touch and the time to enclose the mussel (indicated by a feeding posture; Figures 1B,C) was recorded. Starfish that were feeding after 24 h were included in data analysis and any starfish in the control or test group that had not fed on a mussel after 24 h were discarded from data analysis. The effect of NGFFYamide on feeding behavior was analyzed by a two-tailed Student's *t*-test (time to touch) or Welch's unequal variances *t-*test (time to enclose) using Prism 6 (GraphPad).

#### Investigation of the effects of NGFFYamide on locomotor activity

**Figure 2:**
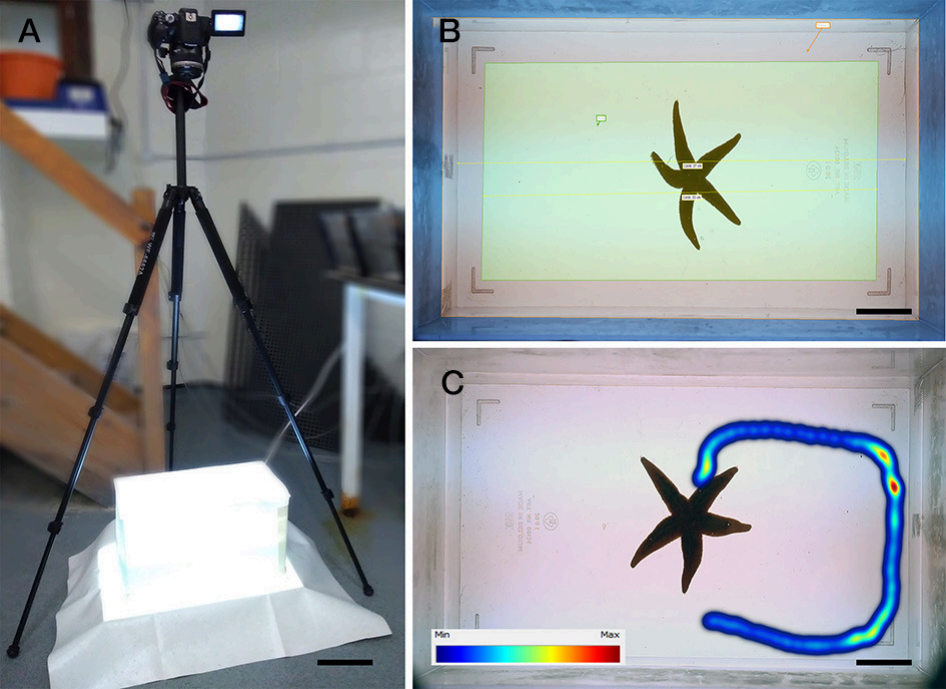
Recording locomotor activity of the starfish *A. rubens* in a laboratory setting. **(A)** Photograph of the experimental set-up, with a camera positioned above a Plexiglas aquarium (37 × 19.8 × 25 cm) containing 7 l of seawater, with all the walls covered with white plastic and situated on top of a lightbox to enhance video contrast, which facilitated detection of the experimental animal by the video-tracking software Ethovision XT1. **(B)** Photograph showing the software arena settings, with the area defined as the floor in yellow (length of 33 cm) and a starfish positioned in the center of the aquarium at the beginning of an experiment. **(C)** Photograph showing tracking of a starfish using Ethovision XT1, with the color scale bar representing the time spent in one position (minimum in blue to maximum in red). Scale bars: **(A)**: 13.1 cm; **(B)**: 4.7 cm; **(C)**: 4.7 cm.

To investigate if NGFFYamide affects locomotor activity in *A. rubens*, 27 adult starfish that had five arms intact and exhibited a normal righting response (59) were selected for testing. A total of 13 animals were injected with distilled water for the control group (mean diameter of 10.6 ± 0.3 cm) and 14 animals were injected with NGFFYamide for the test group (mean diameter 10.4 ± 0.2 cm). Animals in both groups were starved for 1 week to ensure that the feeding status of all animals were similar. The starfish were then transferred to and kept individually in Plexiglas aquaria (27.5 × 19 × 19.6 cm) containing 6 l of aerated seawater and injected with 10 μl of distilled water or 10^−4^ M NGFFYamide, employing the same method as reported above in section 2.5.1. Ten minutes after the injection, starfish were transferred individually to the center of a Plexiglas aquarium (37 × 19.8 × 25 cm) containing 7 l of seawater, with all the walls covered with white plastic and situated on top of a lightbox to enhance video contrast (Figure 2A). Starfish activity was then video recorded from above with a camera (Canon EOS 700D; Figure 2A) for six-and-a-half minutes. Video files (MPEG-4) were then analyzed using the video-tracking software Ethovision XT1 (version 11.5, Noldus; Figures 2B,C). The first 30 s of each six-and-a-half minute video recording were discarded from the analysis to avoid interference caused by starfish handling and surface water movement during these periods. The mean velocity, total distance traveled, maximum acceleration and cumulative time spent on the floor (over an entire 6-min period) as well as mean velocity and total distance traveled (during time intervals of 1-min) were calculated. The effect of NGFFYamide on mean velocity, total distance traveled and cumulative time spent on the floor (over an entire 6-min period) were analyzed using a two-tailed Student's *t-*test. The mean value of maximum acceleration (over an entire 6-min period) and mean velocity and total distance traveled (during the time intervals 1–2 and 3–4 min) were analyzed using a two-tailed Mann-Whitney *U*-test. The mean velocity and total distance traveled (during time intervals of 1-min) in control and NGFFYamide treated groups were analyzed by a Kruskal-Wallis non-parametric test using Dunn's multiple comparisons *post-hoc* test. The Mann-Whitney *U*-test and Kruskal-Wallis non-parametric test were used as these data were found not to be normally distributed by the Kolmogorov-Smirnov normality test. All statistical analyses were carried out using Prism 6 (GraphPad).

## Results

### NGFFYamide is a potent ligand for the *A. rubens* NPS/CCAP-type receptor

**Figure 3:**
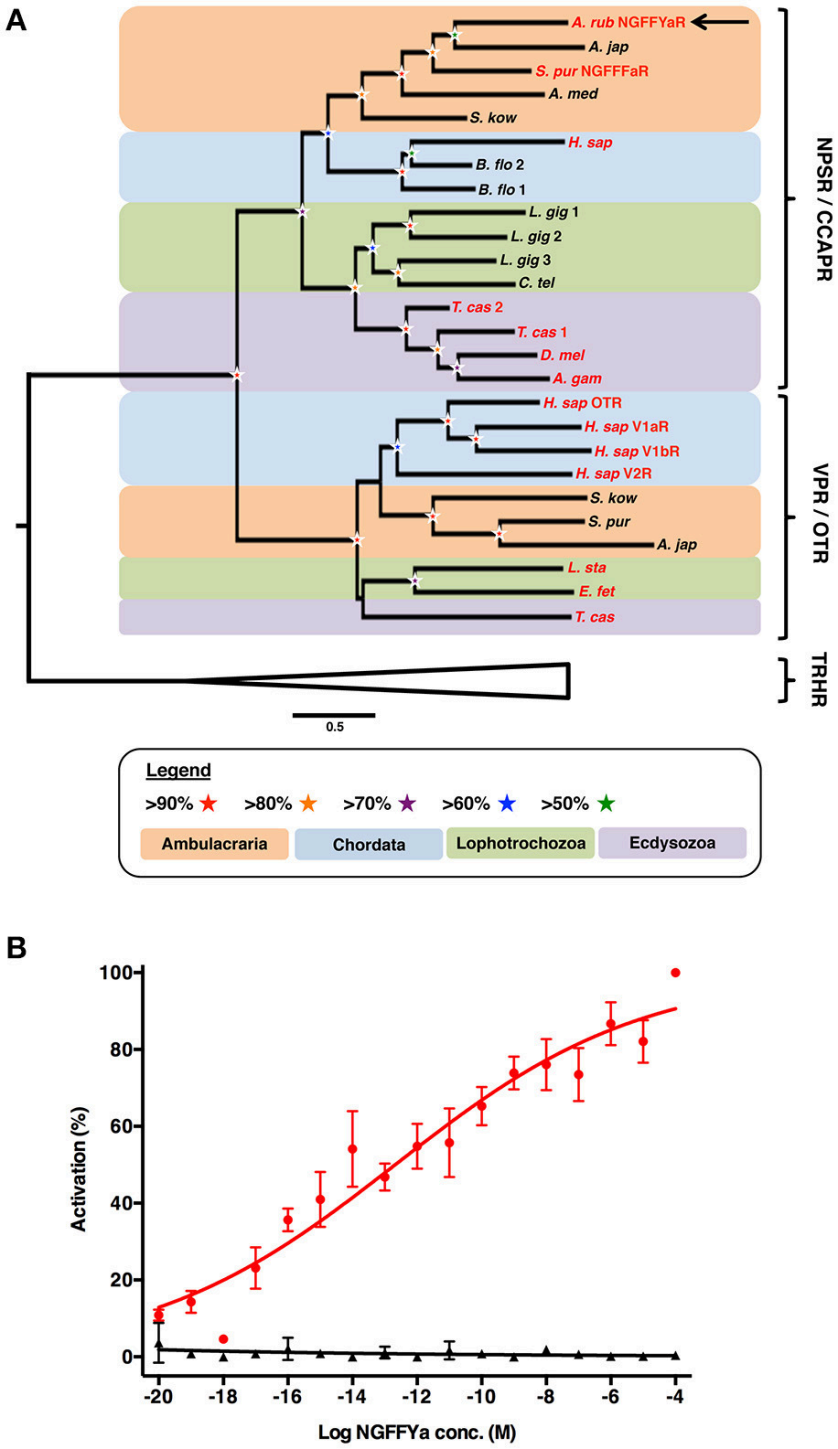
Identification of an NPS/CCAP-type receptor in the starfish *A. rubens* that is activated by the neuropeptide NGFFYamide. **(A)** Phylogenetic tree showing that the *A. rubens* NPS/CCAP-type receptor (arrow) is positioned in a clade comprising NPS/CCAP-type receptors that is distinct from a clade comprising paralogous VP/OT-type receptors. Thyrotropin-releasing hormone (TRH)-type receptors were included here as an outgroup. Bootstrap support (1,000 replicates) for each clade is represented with colored stars as denoted in the key. Species in which the ligands for NPS/CCAP-type receptors and/or VP/OT-type receptors have been identified experimentally are shown with red lettering. Species: *A. gam* (*Anopheles gambiae*); *A. jap* (*Apostichopus japonicus*); *A. med* (*Antedon mediterranea*); *A. rub (Asterias rubens)*; *B. flo* (*Branchiostoma floridae*); *C. tel (Capitella teleta) D. mel (Drosophila melanogaster)*; *E. fet* (*Eisenia fetida*); *H. sap* (*Homo sapiens*); *L. gig (Lottia gigantea)*; *L. sta* (*Lymnaea stagnalis*); *S. kow* (*Saccoglossus kowalevskii*); *S. pur* (*Strongylocentrotus purpuratus*); *T. cas (Tribolium castaneum)*. The accession numbers of sequences included in the phylogenetic tree are listed in Supplementary Table 1. **(B)** Concentration-response curve showing that synthetic NGFFYamide (shown in red) activates the *A. rubens* NPS/CCAP-type receptor in CHO cells co-expressing the promiscuous G-protein Gα16 and over-expressing the GFP-aequorin fusion protein G5A. Control experiments where CHO cells were transfected with an empty pcDNA 3.1(+) vector are shown in black. Each point (± s.e.m.) represents mean values from at least two independent experiments, with each experiment performed in triplicate. Concentration-response data are shown relative (%) to the maximal response (100% activation) observed in each experiment. The EC_50_ value for activation of the NPS/CCAP-type receptor with NGFFYamide is 2.1 × 10^−13^ M.

Phylogenetic analysis of NPS/CCAP-type and paralogous VP/OT-type receptors revealed that the *A. rubens* NPS/CCAP-type receptor groups with ambulacrarian NPS/CCAP-type receptors, including the recently characterized sea urchin (*S. purpuratus*) NGFFFamide receptor (Figure 3A). To determine whether NGFFYamide is the cognate ligand for the *A. rubens* NPS/CCAP-type receptor, the receptor was expressed in a CHO-cell line over-expressing mitochondrially targeted GFP-aequorin fusion protein (G5A) to assay for elevation of calcium (Ca^2+^) levels. To enable this, the receptor was co-transfected with the promiscuous human Gα16 protein, which couples to the phospholipase-C-β (PLC-β) pathway to trigger Ca^2+^ release from intracellular stores. Synthetic NGFFYamide (NGFFY-NH_2_) was tested at concentrations ranging from 10^−20^ to 10^−4^ M and induced concentration-dependent Ca^2+^ elevation, with a half-maximal response concentration (EC_50_) of 2.1 × 10^−13^ M (Figure 3B). Control experiments on cells transfected with an empty pcDNA 3.1 (+) vector revealed no effect of NGFFYamide on Ca^2+^ levels (Figure 3B). Thus, NGFFYamide was identified as a highly potent ligand for the *A. rubens* NPS/CCAP-type receptor.

### Analysis of NGFFYamide precursor expression using mRNA *in situ* hybridization

**Figure 4:**
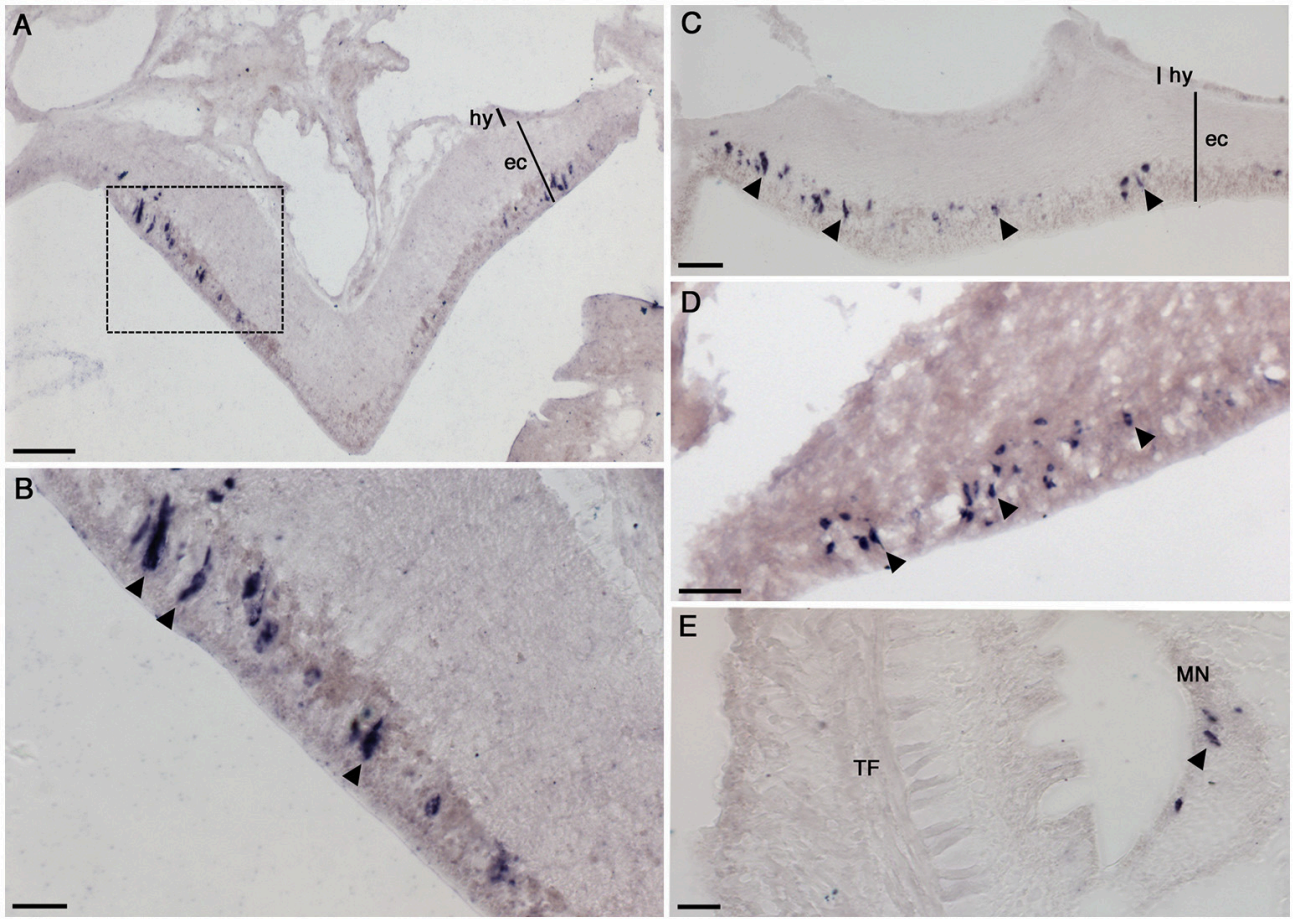
Localization of NGFFYamide precursor mRNA expression in the nervous system of *A. rubens*. **(A)** Transverse section of a radial nerve cord showing stained cells in the epithelium of the ectoneural region, largely concentrated laterally. Note the absence of stained cells in the hyponeural region. The boxed region is shown at a higher magnification in **(B)**, where the elongate, bipolar shape of stained cells (arrowheads) can be seen. **(C)** Longitudinal section of a radial nerve cord showing stained cells (arrowheads) along the length of the epithelium in the ectoneural region (arrowheads). **(D)** Stained cells (arrowheads) in the ectoneural epithelium of the circumoral nerve ring. **(E)** Transverse section of an arm showing stained cells (arrowhead) in the marginal nerve, which is located lateral to the outer row of tube feet on each side of the arm. ec, ectoneural; hy, hyponeural; MN, marginal nerve; TF, tube foot. Scale bars: **(A)**: 33 μm; **(B)**: 16 μm; **(C)**: 16 μm; **(D)**: 33 μm; **(E)**: 16 μm.

**Figure 5:**
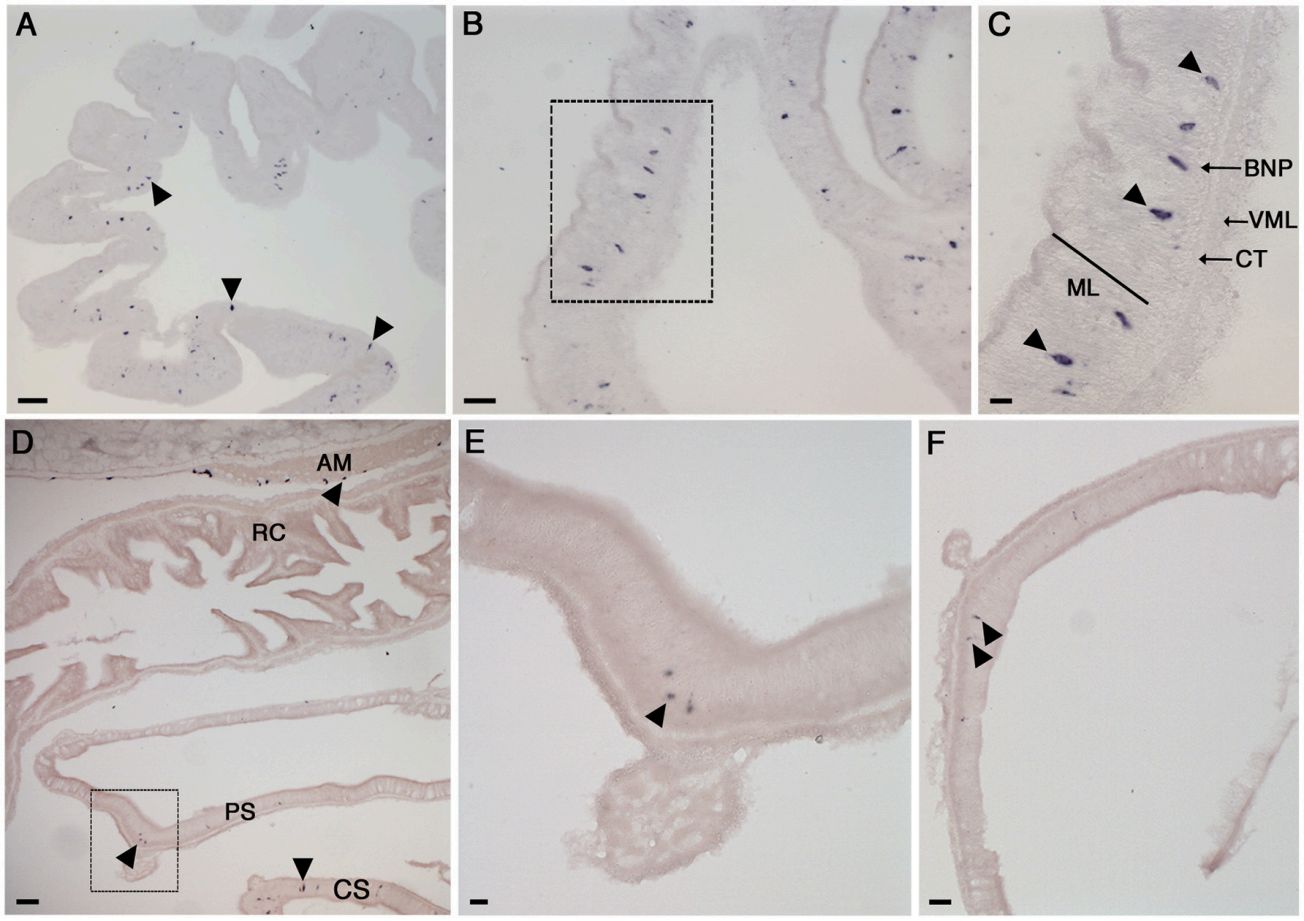
Localization of NGFFYamide precursor mRNA expression in the digestive system of *A. rubens*. **(A)** Low magnification image showing stained cells (arrowheads) widely distributed in a section of a highly folded aboral region of the cardiac stomach. **(B)** Stained cells in the cardiac stomach, with the boxed region shown at a higher magnification in **(C)**, where stained cells (arrowheads) can be seen to be located in the mucosal layer or just beneath the basiepithelial nerve plexus. **(D)** Transverse section of the central disk region showing the cardiac stomach, pyloric stomach, rectal caeca and apical muscle. Stained cells can be seen in the cardiac stomach, pyloric stomach and apical muscle (arrowheads) but not in the rectal caeca. A higher magnification image of expression in the apical muscle is shown in Figure 6E and the boxed region of the pyloric stomach is shown at higher magnification in **(E)**. **(F)** Stained cells (arrowheads) in a transverse section of a pyloric duct. AM, apical muscle; BNP, basiepithelial nerve plexus; CT, collagenous tissue layer; CS, cardiac stomach; ML, mucosal layer; PS, pyloric stomach; RC, rectal caeca; VML, visceral muscle layer. Scale bars: **(A)**: 33 μm; **(B)**: 16 μm; **(C)**: 6 μm; **(D)**: 33 μm; **(E)**: 16 μm; **(F)**: 16 μm.

**Figure 6:**
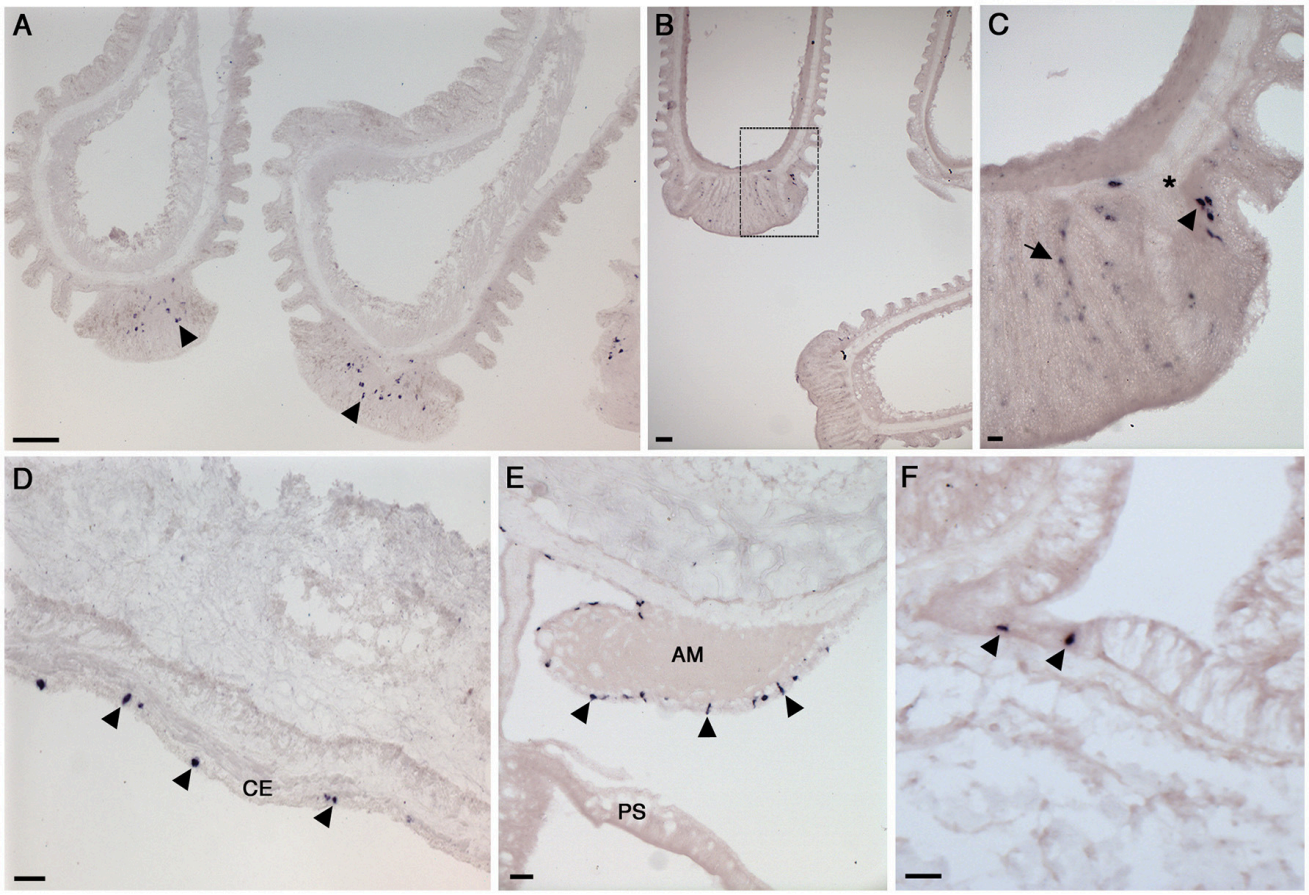
Localization of NGFFYamide precursor mRNA expression in the tube feet, apical muscle and body wall of *A. rubens*. **(A)** Longitudinal sections of two tube feet, showing stained cells (arrowheads) in the sucker region. **(B)** Longitudinal sections of three tube feet, with the boxed region shown at higher magnification in **(C)**; here stained cells (arrowhead) can be seen near to the basal nerve ring (^*^) and in the adhesive region of the tube foot sucker (arrow). **(D)** Transverse section of an arm showing stained cells (arrowheads) in the coelomic epithelium. **(E)** Transverse section at the junction between an arm and the central disk showing stained cells (arrowheads) in the coelomic epithelial lining the apical muscle. **(F)** Transverse section of an arm showing stained cells (arrowheads) in the external epithelium of the body wall. AM, apical muscle; CE, coelomic epithelium; PS, pyloric stomach. Scale bars: **(A)**: 33 μm; **(B)**: 33 μm; **(C)**: 6 μm; **(D)**: 16 μm; **(E)**: 16 μm; **(F)**: 16 μm.

Analysis of the expression of NGFFYamide precursor mRNA transcripts in *A. rubens* [see (61, 62) for detailed descriptions of *A. rubens* anatomy] revealed a widespread pattern of expression. This included stained cells in the nervous system (Figures 4A–E), digestive system (Figures 5A–F), tube feet (Figures 6A–C), coelomic epithelium (Figure 6D), apical muscle (Figure 6E) and body wall (Figure 6F), as described in more detail below.

#### NGFFYamide precursor expression in the nervous system

The *A. rubens* nervous system is comprised of radial nerve cords extending along the oral side of each arm, which are linked in the central disk by a circumoral nerve ring. Marginal nerves run parallel with the radial nerve cords, lateral to the outer row of tube feet on each side of the arm. Analysis of NGFFYamide precursor mRNA expression revealed stained cells in the ectoneural region of the radial nerve cords; no stained cells were observed in the hyponeural region of the radial nerve cords (Figures 4A–C). The labeled cell bodies were, largely, laterally concentrated at the periphery of the radial nerve cord in the epithelial layer of the ectoneural region (Figures 4A–C). Expression of NGFFYamide precursor mRNA was also observed in the ectoneural region of the circumoral nerve ring (Figure 4D), consistent with the expression pattern observed in the radial nerve cords. Furthermore, NGFFYamide precursor mRNA expression was also observed in the marginal nerves (Figure 4E).

#### NGFFYamide precursor expression in the digestive system

The oral region of the *A. rubens* digestive system comprises a mouth surrounded by a contractile peristomial membrane, which is continuous with a short tubular esophagus. The esophagus is linked aborally to a large and highly folded cardiac stomach, which is everted through the mouth during feeding. The pyloric stomach is aboral to the cardiac stomach and is linked to pyloric caeca via pyloric ducts. Aboral to the pyloric stomach is a short rectum, which has associated rectal caeca. Analysis of NGFFYamide precursor mRNA expression revealed stained cells in the cardiac stomach (Figures 5A–C) with sparser expression detected in the pyloric stomach and very sparse expression detected in the pyloric ducts (Figures 5D–F). Expression of NGFFYamide mRNA was not observed in the peristomial membrane, esophagus, pyloric caecae or rectal caeca (data not shown).

Labeled cells were more densely concentrated in the aboral region of the cardiac stomach than in the oral region of the cardiac stomach and were located in the mucosal layer, often in close proximity to the basiepithelial nerve plexus (Figures 5A–C). Labeled cells were sparsely distributed in both the pyloric stomach and pyloric ducts, where they were located in the mucosal layer in close proximity to the basiepithelial nerve plexus (Figures 5D–F).

#### NGFFYamide precursor expression in tube feet, apical muscle, and body wall

The body wall skeleton is comprised of calcite ossicles that are interconnected by muscle and collagenous tissue. The aboral surface of the body wall has a number of appendages, including pedicellariae, spines, and papulae. The oral surface of the body wall comprises ambulacral and adambulacral ossicles and two rows of tube feet, which are linked to ampullae that are located internally. Analysis of NGFFYamide precursor mRNA expression revealed stained cells in the sucker region of tube feet (Figures 6A–C), with some stained cells closely associated with the basal nerve ring (Figures 6A–C).

NGFFYamide precursor mRNA expression was observed in cells located in the coelomic epithelium, which lines the internal surface of the body wall (Figure 6D). Stained cells were also observed in the coelomic epithelial lining of the apical muscle, an aboral thickening of longitudinally oriented muscle in the arm body wall that also extends into the central disk region (Figure 6E). Finally, a sparse population of stained cells were observed in the external epithelium of the body wall (Figure 6F).

### NGFFYamide causes relaxation of *in vitro* preparations of apical muscle and induction of tonic and phasic contraction of tube feet

**Figure 7:**
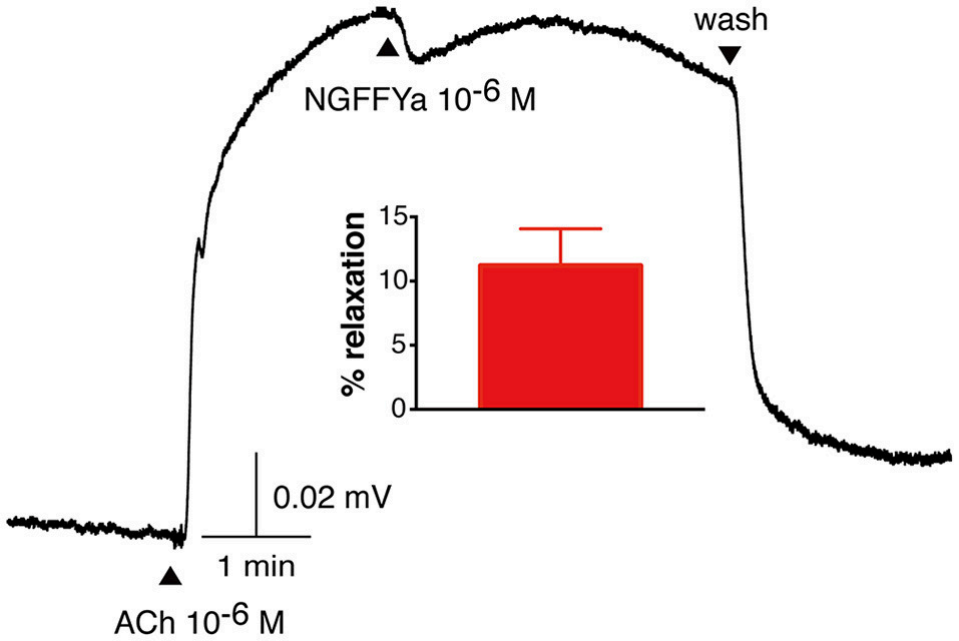
NGFFYamide causes relaxation of *in vitro* acetylcholine-contracted apical muscle preparations from *A. rubens*. A representative recording is shown, with 10^−6^ M NGFFYamide causing transient partial reversal of the contracting action 10^−6^ M acetylcholine (ACh). Application of ACh and NGFFYamide are labeled with upward pointing arrowheads and washing of the preparation is labeled with a downward pointing arrowhead. The graph shows the mean (± s.e.m.) relaxant effect of NGFFYamide when tested at a concentration of 10^−6^ M (*n* = 4).

NGFFYamide caused relaxation of ACh-contracted apical muscle preparations when tested at concentrations of 10^−6^ and 10^−5^ M *in vitro*. However, the mean relative relaxing effect of NGFFYamide at 10^−6^ M was quite small, amounting to only an 11.3 ± 2.9 % reversal of the contracting effect of ACh at 10^−6^ M (Figure 7; *n* = 4). Furthermore, when tested at the higher concentration of 10^−5^ M, a similar modest relaxing effect of NGFFYamide was observed (12.1 ± 7.8 %; data not shown; *n* = 4). The relaxing effect of NGFFYamide was observed when the apical muscle was contracted with 10^−6^ M ACh but NGFFYamide seems to have no effect on apical muscle preparations contracted with 10^−5^ M ACh or when the apical muscle was not pre-contracted with ACh (data not shown; *n* = 4).

**Figure 8:**
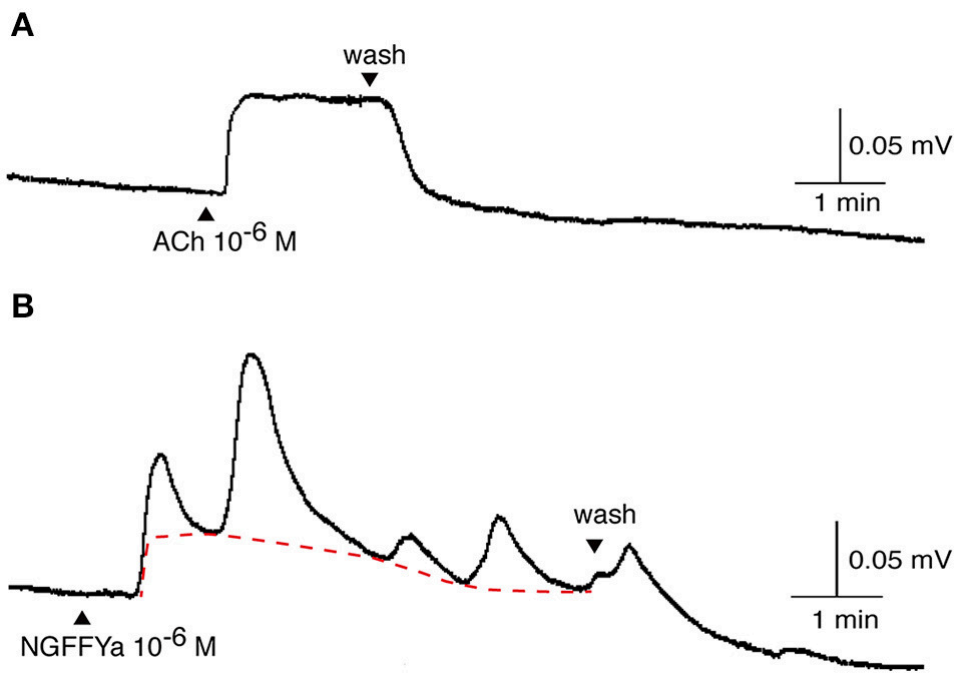
Application of NGFFYamide to *in vitro* tube foot preparations from *A. rubens* triggers phasic contractions that are superimposed upon an increase in basal tone. Representative recordings from a single tube foot preparation are shown, comparing the effect of **(A)** acetylcholine (ACh; 10^−6^ M) and the effect of **(B)** NGFFYamide (10^−6^ M). Application of ACh and NGFFYamide are labeled with upward pointing arrowheads and washing of the preparation is labeled with a downward pointing arrowhead.

**Figure 9:**
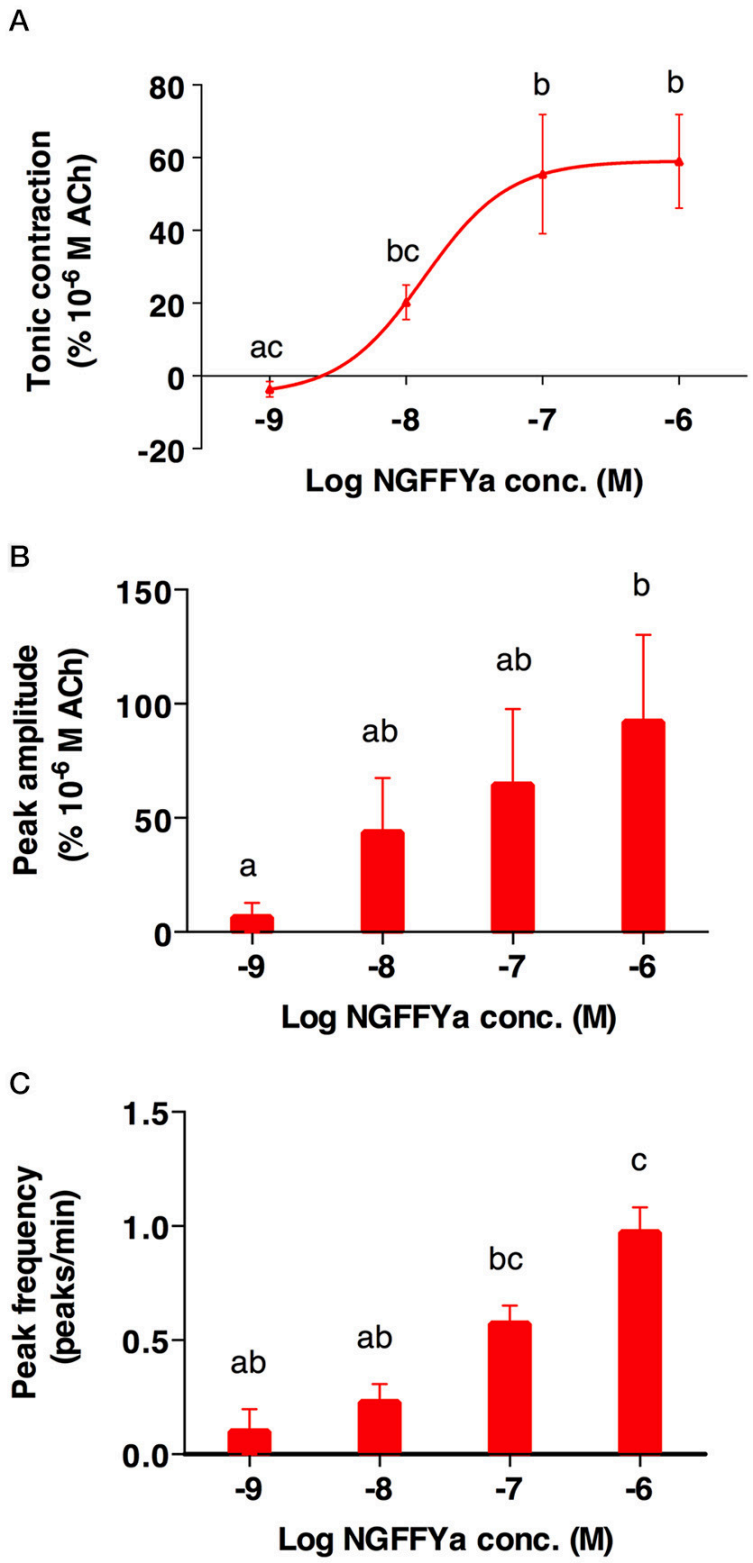
The effects of NGFFYamide in causing both tonic and phasic contraction of tube foot preparations from *A. rubens* are concentration dependent. **(A)** Concentration-dependence of the effect of NGFFYamide in causing tonic contraction of tube feet. **(B)** Concentration-dependence of the effect of NGFFYamide on the peak amplitude of NGFFYamide-induced phasic contractions. **(C)** Concentration-dependence of the effect of NGFFYamide on the peak frequency of NGFFYamide-induced phasic contractions. The effects of NGFFYamide shown in **(A,C)** are normalized to the contractile effect observed with 10^−6^ M Acetylcholine (ACh). Results are expressed as mean ± s.e.m. (*n* = 6). Different letters indicate statistically significant differences (*p* < 0.05) by one-way ANOVA (A) or Kruskal-Wallis test **(B,C)**.

NGFFYamide had an excitatory effect on *in vitro* preparations of tube feet, triggering phasic contractions that were superimposed on an increase in basal tone (Figures 8, 9). Thus, NGFFYamide caused concentration-dependent tonic contraction of tube foot preparations at concentrations between 10^−8^ and 10^−6^ M (*n* = 6; Figure 9A). The mean maximal contractile effect, 59.1 ± 12.5 % in comparison with the effect of ACh at 10^−6^ M (defined as 100 % contraction), was achieved at a concentration of 10^−6^ M (Figure 9A). The half maximal response concentration (EC_50_) was 1.4 × 10^−8^ M. NGFFYamide typically induced concentration-dependent phasic contractions of tube feet preparations between 10^−8^ and 10^−6^ M, although phasic contractions were induced at 10^−9^ M in one preparation (Figures 8, 9B,C). The mean maximal peak frequency (0.97 ± 0.11) and the mean maximal peak amplitude (70.9 ± 28.2 %) were achieved at a NGFFYamide concentration of 10^−6^ M (Figures 9B,C).

It is noteworthy that the potency of NGFFYamide as a ligand for its receptor when the receptor is over-expressed heterologously in CHO-K1 cells *in vitro* (EC_50_ = 2.1 × 10^−13^ M; Figure 3B) is much higher than its potency *in vitro* as a regulator of the contractility of cardiac stomach (39), apical muscle (Figure 7), and tube foot (Figures 8, 9) preparations. This probably reflects both the non-physiological over-expression of the NGFFYamide receptor in CHO-K1 cells and the reduced access of NGFFYamide to receptors in muscle/organ preparations caused by impaired penetration through tissue layers and degradation of the peptide by endogenous peptidases.

### NGFFYamide inhibits feeding behavior in *A. rubens*

Based on the anatomical expression of the NGFFYamide precursor transcript in the digestive system using mRNA *in situ* hybridization and the previously reported effect of NGFFYamide on the cardiac stomach both *in vitro* and *in vivo* (39), we hypothesized that NGFFYamide may affect feeding behavior in *A. rubens*.

**Figure 10:**
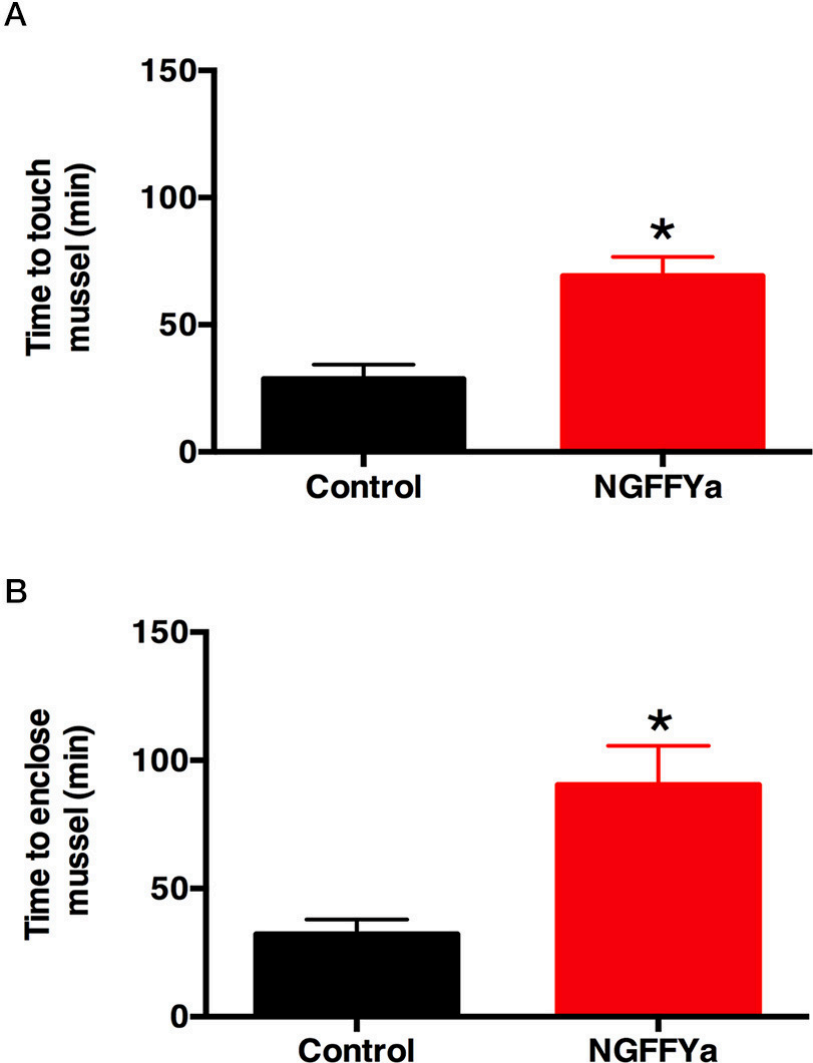
Effects of *in vivo* injection of NGFFYamide on feeding behavior in *A. rubens*. **(A)** Injection of NGFFYamide (10 μl of 10^−4^ M NGFFYamide; shown in red) causes a significant increase in the time elapsed (minutes) before starfish touch a mussel (see Figure 1A), by comparison with control or vehicle-injected (10 μl of distilled water; shown in black) animals. **(B)** Injection of NGFFYamide (10 μl of 10^−4^ M NGFFYamide; shown in red) causes a significant increase in the time elapsed (minutes) before starfish enclose a mussel (see Figures 1B,C), by comparison with control or vehicle-injected (10 μl of distilled water; shown in black) animals. Data are expressed as mean ± s.e.m (*n* = 15 for control group; *n* = 17 for NGFFYamide-treated group). ^*^Statistically significant differences (*p* < 0.005) between vehicle-injected and the NGFFYamide-treated groups, as determined by student *t*-test **(A)** or Welch's *t*-test **(B)**.

Injection of starfish with NGFFYamide (10 μl of 10^−4^ M NGFFYamide) caused a significant increase in the time for starfish to make first contact with a mussel (“time to touch”; *p* < 0.0005; Figure 10A) and in the time to adopt a feeding posture (“time to enclose”; *p* < 0.005; Figure 10B). During the 5-h observation period, five NGFFYamide-treated starfish touched the mussel two times before enclosing. One starfish from the control group was discarded from the analysis, as it had atypically not fed on a mussel during the 24 h after treatment.

### NGFFYamide causes a reduction in locomotor activity in *A. rubens*

Based on the expression of the NGFFYamide precursor transcript in tube feet, the effect of NGFFYamide on *in vitro* tube foot preparations (see section NGFFYamide causes relaxation of *in vitro* preparations of apical muscle and induction of tonic and phasic contraction of tube feet) and the increased time to initiate feeding (see section NGFFYamide inhibits feeding behavior in *A. rubens*), we hypothesized that NGFFYamide may affect locomotor activity in *A. rubens*.

**Figure 11:**
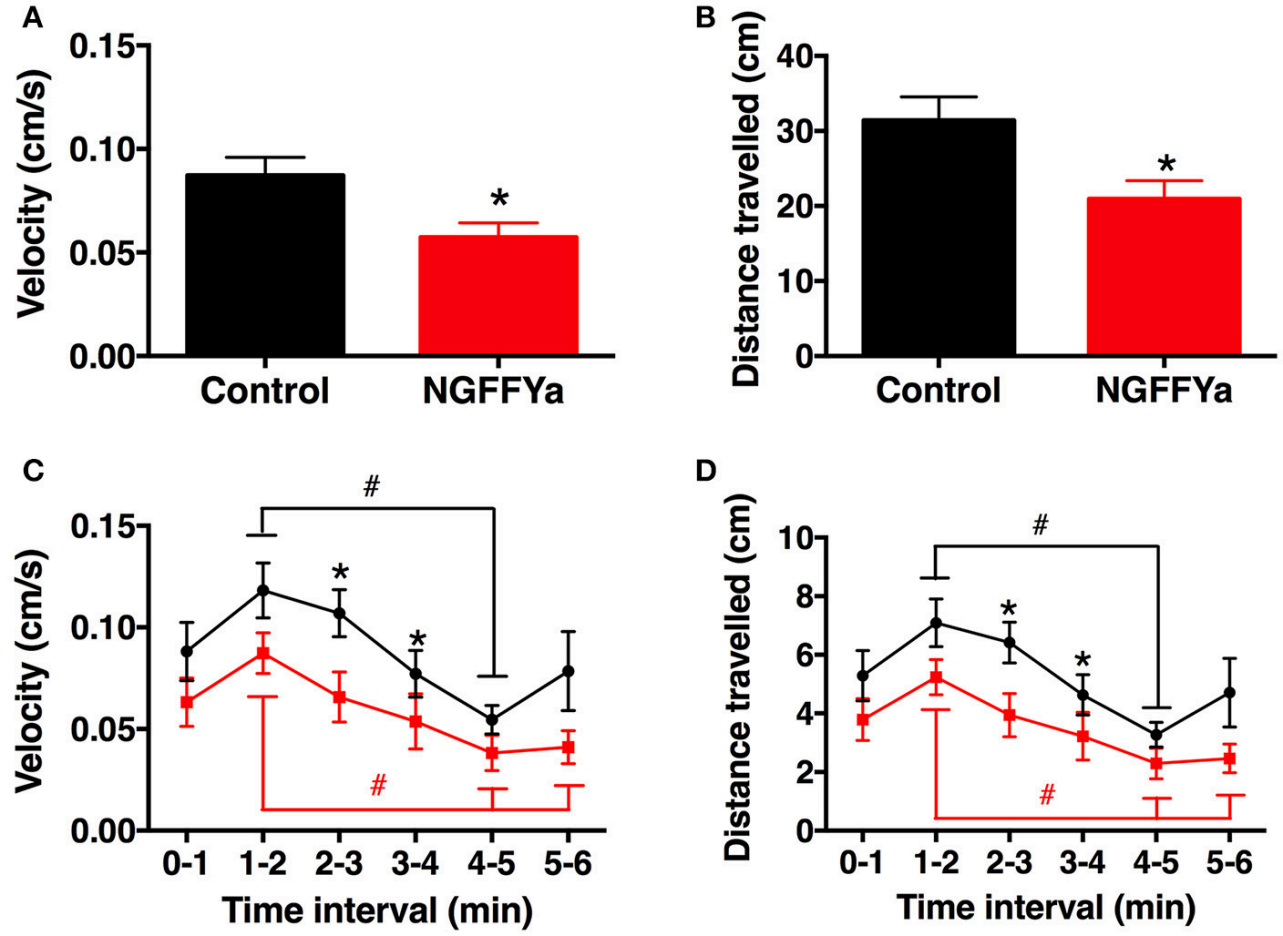
Effects of *in vivo* injection of NGFFYamide on locomotor activity in *A. rubens*. Graphs showing **(A)** mean velocity (cm/s) and **(B)** distance traveled (cm) over an entire 6-min period, between ten-and-half minutes to sixteen-and-a-half minutes after injection with vehicle (10 μl of distilled water; control group; shown in black) or NGFFYamide (10 μl of 10^−4^ M NGFFYamide; shown in red). Graphs showing **(C)** mean velocity (cm/s) and **(D)** distance traveled (cm) during time intervals of 1-min over a 6-min experiment, between ten-and-half minutes to sixteen-and-a-half minutes after injection with vehicle (10 μl of distilled water; control group; shown in black) or NGFFYamide (10 μl of 10^−4^ M NGFFYamide; shown in red). Data are expressed as mean ± s.e.m (*n* = 13 for control group; *n* = 14 for NGFFYamide-treated group). ^*^Statistically significant differences (*p* < 0.05) between control and NGFFYamide-treated groups, as determined by Student's *t*-test **(A,B)** or Mann-Whitney *U*-test **(C,D)**, and #statistically significant differences determined by Kruskal-Wallis test within each group (black for control- and red for NGFFYamide-treated group; *p* < 0.005).

*In vivo* administration of NGFFYamide (10 μl of 10^−4^ M NGFFYamide) caused a significant decrease in the mean velocity of locomotor activity (Figure 11A) and the total distance traveled (Figure 11B) for the period of time from ten-and-half minutes to sixteen-and-a-half minutes after treatment (*p* < 0.05). NGFFYamide treatment did not affect the maximum acceleration (10.1 ± 4.4 cm/s^2^ for control-and 5.7 ± 1.6 cm/s^2^ for NGFFYamide-treated group) or the cumulative time spent on the floor (169.6 ± 65.6 s for control and 211 ± 22.2 s for NGFFYamide-treated group) during the same time period. Furthermore, analysis of activity during each 1-min period of the 6-min experiment revealed significant differences between control and NGFFYamide-treated groups (*p* < 0.05) at time points from 2-to-3 min and from 3-to-4 min (Figures 11C,D).

## Discussion

Here, we report the characterization of a NG peptide-type neuropeptide signaling system in the starfish *A. rubens*. Our demonstration that the amidated pentapeptide NGFFYamide acts as a ligand for an *A. rubens* NPS/CCAP-type receptor is consistent with our previously reported discovery that the structurally similar neuropeptide NGFFFamide acts as a ligand for an NPS/CCAP-type receptor in the sea urchin *S. purpuratus* (8). Collectively, these findings provide definitive proof that NG peptides in echinoderms are orthologs of NPS in vertebrates and CCAP in protostomes. Furthermore, the discovery of the NGFFYamide signaling system in *A. rubens* has provided a basis for (*i)* investigation of the physiological roles of NGFFYamide in starfish, (*ii)* comparison of the actions of NGFFYamide with what is known about the actions of NG peptides in other echinoderms and (*iii)* comparison of the actions of NPS/NG peptide/CCAP-type neuropeptides in different phyla, as discussed below.

### Functional characterization of NGFFYamide signaling in the starfish *A. rubens*

We reported previously that NGFFYamide causes dose-dependent contraction of cardiac stomach preparations from *A. rubens in vitro* and triggers retraction of the everted cardiac stomach when administered *in vivo* (39). Here, our observation that the NGFFYamide precursor transcript is detected in cells located in the mucosa or basiepithelial nerve plexus layer of the cardiac stomach is consistent with these pharmacological effects of NGFFYamide. Furthermore, these findings indicate that NGFFYamide may be released by cells in the cardiac stomach to regulate its contractile state physiologically. The context in which NGFFYamide is released by cells in the cardiac stomach remains to be determined. However, plausible scenarios are that NGFFYamide is released physiologically to trigger retraction of the cardiac stomach when extraoral feeding on prey is completed and/or to trigger premature retraction of the cardiac stomach if predators or other external stressors are sensed during a feeding bout. Informed by these observations and conclusions, here we investigated if *in vivo* injection NGFFYamide affects the normal feeding behavior of starfish. Interestingly, we found that intracoelomic injection of NGFFYamide caused a significant increase in the time taken to make contact with prey (a mussel) and a significant increase in the time taken to enclose a mussel as a prelude to extraoral feeding. The physiological relevance of this effect of NGFFYamide remains to be elucidated and further insights may be obtained if the location of NGFFYamide receptors in *A. rubens* can be determined. Nevertheless, one possibility is that a direct effect of NGFFYamide on the contractile state of the cardiac stomach and/or a direct effect of NGFFYamide on the nervous system inhibits appetitive behavior of starfish. Another possibility is that the effect of NGFFYamide on feeding behavior is an indirect consequence of effects of NGFFYamide on locomotor systems and locomotor activity in starfish. Therefore, here we also investigated the *in vitro* effects of NGFFYamide on organs and muscle systems associated with locomotor activity in starfish and the *in vivo* effects of NGFFYamide on locomotor activity in starfish, as discussed below.

Our analysis of the anatomical distribution of the NGFFYamide precursor transcript in *A. rubens* revealed expression not only in the central nervous system (radial nerve cords, circumoral nerve ring) and digestive system, but also in other organs. Thus, NGFFYamide precursor-expressing cells are present in the coelomic lining of the apical muscle, a thickening of the longitudinally orientated body wall muscle layer located in an aboral and sagittal position in each arm. Investigation of the *in vitro* effect of NGFFYamide on apical muscle preparations revealed that it causes relaxation, partially reversing the contracting action of ACh. However, the effect of NGFFYamide on the apical muscle was quite modest. Thus, NGFFYamide caused only an 11–12% reversal of the contraction induced by 10^−6^ M ACh, when tested at concentrations of 10^−6^ or 10^−5^ M. Furthermore, at these concentrations NGFFYamide had no effect on preparations contracted with 10^−5^ M ACh. By way of comparison, other neuropeptides are more effective as relaxants of the apical muscle preparation. Thus, when tested at a concentration of 10^−6^ M, the SALMFamide-type neuropeptide S2, the pedal peptide-type neuropeptide ArPPLN1b and the calcitonin-type neuropeptide ArCT cause ~8, ~16, and ~40 % reversal of contraction induced by application of 10^−5^ M ACh to apical muscle preparations (53, 63). Nevertheless, the discovery that NGFFYamide acts as a relaxant of the apical muscle adds to a growing list of neuropeptides that regulate the contractile state of this preparation, which also include neuropeptides that cause contraction of the apical muscle in *A. rubens*—the gonadotropin-releasing hormone type neuropeptide ArGnRH and the corazonin-type neuropeptide ArCRZ (54).

Consistent with the expression of the NGFFYamide precursor transcript in cells associated with the locomotory tube feet of starfish, we found that NGFFYamide had an excitatory effect on *in vitro* preparations of tube feet. Thus, application of NGFFYamide triggered phasic contractions of tube feet that were superimposed upon an increase in basal tone. Furthermore, there was concentration-dependency in the effect of NGFFYamide on both the amplitude and the frequency of the phasic contractions. This action of NGFFYamide on tube feet is interesting because it is the first neuropeptide that we have found to have this type of excitatory effect on tube feet. Thus, we have reported previously that the neuropeptides ArGnRH and ArCRZ cause dose-dependent tonic contraction of tube foot preparations (54), whereas NGFFYamide is so far unique amongst the neuropeptides that have been tested on starfish tube foot preparations in inducing initiation of phasic contractions. This effect of NGFFYamide suggests that it acts to induce activation of an endogenous motor rhythm generator and therefore NGFFYamide may be important physiologically in regulating the co-ordinated stepping action of tube feet when starfish move. Informed by these observations, we investigated the effect of *in vivo* injection of NGFFYamide on locomotor activity of starfish and, interestingly, we found that NGFFYamide causes a significant reduction in velocity and the distance traveled. How can we reconcile what at first sight appear to be inconsistent effects of NGFFYamide at the level of individual tube feet *in vitro* and whole-animals *in vivo*? If, as suggested above, NGFFYamide participates in neural mechanisms that regulate initiation and/or maintenance of the rhythmic stepping action of tube feet, then a consequence of *in vivo* injection of NGFFYamide may be a pharmacological disruption of finely tuned endogenous neurochemical and electrophysiological tube foot control systems. Therefore, the inhibitory effect of NGFFYamide on whole-animal locomotor activity is not necessarily indicative of the physiological role of this neuropeptide. Nevertheless, in combination, the *in vitro* effects of NGFFYamide on tube feet and the *in vivo* effect of NGFFYamide on locomotor activity indicate that NGFFYamide has a physiological role in regulation of tube foot and locomotor activity in starfish.

Discovery of the inhibitory effect of NGFFYamide on locomotor activity may also have relevance to the inhibitory effect of NGFFYamide on feeding behavior. Our assessment of the effect of NGFFYamide on feeding behavior in starfish involved measurement of the time taken to make contact with prey and the time taken to adopt a feeding posture and therefore the inhibitory effect of NGFFYamide on these activities could simply be manifestations of a general inhibitory effect on locomotor activity. Therefore, it remains to be established whether or not NGFFYamide actually has a direct effect on the initiation of feeding behavior in starfish. Nevertheless, the previously reported powerful effect of NGFFYamide in triggering cardiac stomach contraction and retraction are strong indicators that this neuropeptide may be important physiologically in mediating termination of feeding activity in starfish.

### Comparative analysis of the physiological roles of NG peptides in echinoderms

Having analyzed both the anatomical expression pattern and bioactivity of NGFFYamide in the starfish *A. rubens*, it is of interest to make comparisons with what is known about the physiological roles of NG peptides in other echinoderms. The first NG peptide to be identified in an echinoderm was NGIWYamide, which was isolated from the sea cucumber *A. japonicus* on account of its effect in causing contraction of longitudinal body wall muscles in this species (42). Subsequently, NGFFFamide was found to cause contraction of tube foot and esophagus preparations from the sea urchin *E. esculentus* (35) and NGFFYamide was found to cause contraction of cardiac stomach preparations from the starfish *A. rubens* (39). However, NG peptides do not only act to cause tonic contraction of muscle preparations in echinoderms. This has been illustrated in this study with the finding that NGFFYamide causes relaxation of ACh-contracted apical muscle preparations and triggers phasic contractions of tube foot preparations. Accordingly, analysis of the *in vitro* pharmacological effects of NGIWYamide in *A. japonicus* has similarly revealed differing actions on different preparations from this species. Thus, whilst NGIWYamide induces tonic contraction of both longitudinal body wall muscle and tentacle preparations, it also causes inhibition of spontaneous phasic contractions of intestine preparations (43). Thus, in both starfish and sea cucumbers, NG peptides have myoexcitatory or myoinhibitory effects. However, the physiological significance and mechanisms of the differing actions of NG peptides on different muscle preparations from starfish and sea cucumbers remain to be determined. Furthermore, the actions of NGIWYamide are not restricted to effects on muscle activity as this peptide also causes stiffening of body wall mutable collagenous tissue (64) and induction of oocyte maturation and spawning (44) in *A. japonicus*. Thus, we can conclude that NG peptides are pleiotropic neuropeptides in echinoderms and it is likely that other physiological roles of NG peptides in echinoderms will be discovered if their actions are further investigated using a variety of bioassays.

### Comparison of physiological roles NPS/NG peptide/CCAP-type neuropeptides: conservation or diversification of neuropeptide function?

The functional characterization of echinoderm NG peptides that are orthologs of NPS-type neuropeptides in vertebrates and CCAP-type neuropeptides in protostomes has provided a basis for comparison of the physiological roles of NPS/NG peptide/CCAP-type neuropeptides and investigation of the evolution of neuropeptide function in this neuropeptide family. To facilitate this, it is necessary to first provide a brief overview of what is known about the physiological roles of NPS-type neuropeptides in vertebrates and CCAP-type neuropeptides in protostomes.

NPS was first discovered as a constituent of brain extracts that is the endogenous ligand for the “orphan” G-protein coupled receptor GPR154 (65). Thus, nothing was known about its physiological roles when NPS was first identified. Functional characterization of NPS revealed that it increases locomotor activity in mice, increases wakefulness in rats and has anxiolytic-like effects in mice exposed to stressful conditions (9), actions that have been confirmed and examined in more detail in subsequent studies on rodents (16–19, 66–70). Furthermore, evidence that the anxiolytic effect of NPS in rodents is also applicable to humans has emerged with the discovery that a coding polymorphism in the NPSR is associated with panic disorder (13, 14). Roles of NPS in regulation of other physiological/behavioral processes in mammals have also been revealed, including immune cell activity and asthma susceptibility (71), irritable bowel syndrome/inflammatory bowel disease (22) and inhibition of food intake (15, 18, 72–75). However, interestingly, one study has revealed that NPS can increase food intake in rodents, which may occur through the activation of orexin-expressing neurons (76).

Turning to CCAP, this neuropeptide was first discovered as a cardioexcitatory peptide in crustaceans (25) and subsequent studies have revealed cardioexcitatory effects of CCAP-type neuropeptides in insects (75, 77). Furthermore, there is substantial evidence that CCAP is one of several neuropeptides that regulate shedding of the exoskeleton (ecdysis) in arthropods. Thus, a dramatic increase in circulating levels of CCAP has been observed in two crustacean species during ecdysis (26). Furthermore, CCAP activates an ecdysis motor program in the moth *Manduca sexta* (28, 78, 79), ablation of CCAP-expressing neurons results in failure of pupal ecdysis in *Drosophila melanogaster* (80, 81) and reducing expression of CCAP and its receptor by RNA interference causes failure of ecdysis in the beetle *Tribolium castaneum* (31, 75). Other actions of CCAP in insects that have been reported include induction of oviduct contractions in the locust *Locusta migratoria* (82), inhibition of locomotor activity in the cockroach *P. americana* (33) and stimulation of gut contraction and secretion of digestive enzymes in cockroaches (32, 83). Thus, CCAP is a pleiotropic neuropeptide in insects, with roles in regulation of a variety of physiological/behavioral processes. Currently, very little is known about the physiological roles of CCAP-type neuropeptides in non-arthropodan protostomes. However, evidence of roles in regulation of feeding behavior in the mollusc (pond snail) *Lymnaea stagnalis* (84) and regulation of egg-laying in the mollusc (cuttlefish) *Sepia officinalis* (85) has been reported.

Do any common themes emerge from this overview of the actions of NPS/CCAP-type neuropeptides in vertebrates and protostomes by way of comparison with the actions of NG peptides in echinoderms? Clearly, there are huge differences in the anatomical organization of the nervous systems, digestive systems and muscle systems of vertebrates, echinoderms and protostomes, which complicate comparisons of neuropeptide function. Nevertheless, it remains of interest to identify any similarities/differences at the level of physiological processes and behavior. Effects on locomotor activity recur, with both stimulatory (rodents) and inhibitory (cockroaches, starfish) actions reported. Likewise, effects on the digestive system and on feeding behavior have been reported in several taxa, including stimulation (cockroaches, starfish) or inhibition (sea cucumbers) of gut contractile activity and stimulation (pond snail, rodents) or inhibition (rodents, chicks, starfish) of feeding or feeding-related processes. Evidence of roles in regulation of reproductive processes also recur, with NGIWYamide triggering spawning in sea cucumbers and CCAP-type neuropeptides linked with egg-laying in locusts and cuttlefish. Thus, there is evidence of both evolutionary conservation and diversification in the functions of NPS/NG peptide/CCAP-type neuropeptides. More investigations of the actions of this family of neuropeptides in a wider range of taxa, including other echinoderms and non-arthropodan protostomes, are now needed to gain deeper insights into the evolution of the physiological roles of NPS/NG peptide/CCAP-type neuropeptides in the Bilateria.

## Ethics statement

Approval by the local institution/ethics committee was not required for this work because experimental work on starfish is not subject to regulation.

## Author contributions

DS phylogenetic analysis of NPS/CCAP-type receptors. DS pharmacological characterization of the NGFFYamide receptor. DS, EP, and ME mRNA *in situ* hybridization study. AT *in vitro* pharmacological studies. AT and EG *in vivo* pharmacological analysis of the effect of NGFFYamide on feeding behavior. AT *in vivo* pharmacological analysis of the effect of NGFFYamide on locomotion. MRE, AT, and DS conceived the study and wrote the first draft of the paper. All authors contributed to the submitted final draft of the paper and gave final approval for publication.

### Conflict of interest statement

The authors declare that the research was conducted in the absence of any commercial or financial relationships that could be construed as a potential conflict of interest.
